# The Mediterranean scorpion *Mesobuthus gibbosus* (Scorpiones, Buthidae): transcriptome analysis and organization of the genome encoding chlorotoxin-like peptides

**DOI:** 10.1186/1471-2164-15-295

**Published:** 2014-04-21

**Authors:** Elia Diego-García, Figen Caliskan, Jan Tytgat

**Affiliations:** 1 Toxicology and Pharmacology, University of Leuven, Campus Gasthuisberg O& N2,PO Box 922, Herestraat 49, 3000 Leuven, Belgium; 2 Department of Biology, Faculty of Science and Art, Eskisehir Osmangazi University, Campus Meselik, 26, 480 Eskisehir, Turkey

**Keywords:** Scorpion transcriptome, Chlorotoxin, Genomic organization, Mesobuthus gibbosus, Venom glands, Scorpion toxin

## Abstract

**Background:**

Transcrof toxin genes of scorpion species have been published. Up to this moment, no information on the gene characterization of *M. gibbosus* is available.

**Results:**

This study provides the first insight into gene expression in venom glands from *M. gibbosus* scorpion. A cDNA library was generated from the venom glands and subsequently analyzed (301 clones). Sequences from 177 high-quality ESTs were grouped as 48 Mgib sequences, of those 48 sequences, 40 (29 “singletons” and 11 “contigs”) correspond with one or more ESTs. We identified putative precursor sequences and were grouped them in different categories (39 unique transcripts, one with alternative reading frames), resulting in the identification of 12 new toxin-like and 5 antimicrobial precursors (transcripts). The analysis of the gene families revealed several new components categorized among various toxin families with effect on ion channels. Sequence analysis of a new KTx precursor provides evidence to validate a new KTx subfamily (α-KTx 27.x). A second part of this work involves the genomic organization of three Meg-chlorotoxin-like genes (ClTxs). Genomic DNA sequence reveals close similarities (presence of one same-phase intron) with the sole genomic organization of chlorotoxins ever reported (from *M. martensii*).

**Conclusions:**

Transcriptome analysis is a powerful strategy that provides complete information of the gene expression and molecular diversity of the venom glands (telson). In this work, we generated the first catalogue of the gene expression and genomic organization of toxins from *M. gibbosus.* Our result represents a relevant contribution to the knowledge of toxin transcripts and complementary information related with other cell function proteins and venom peptide transcripts. The genomic organization of the chlorotoxin genes may help to understand the diversity of this gene family.

## Background

The evolutionary history of the scorpions begun around 425–450 million years ago, in the middle Silurian [1] and these animals are therefore often considered “living fossils”. Scorpions are morphologically conservative organisms [2] and approximately 1500 species are recognized and classified in different families [1,3]. The family Buthidae is geographically distributed worldwide and is the largest of the scorpion families, comprising 81 genera and 570 species [3]. Moreover, from a clinical perspective, Buthidae is the most important scorpion family [4]. Several members of this family are toxic to mammals and can be dangerous to humans [3]. Stings by scorpion species dangerous to humans can induce different levels of toxicity and sometimes have lethal consequences. Scorpion venom consists of a mixture of biologically active compounds: (poly-) peptide toxins that specifically target ion channels (Na^+^, Cl^−^, K^+^ and Ca^2+^) and other cellular receptors [5]. In terms of venom, scorpion biodiversity is reflected in more than 134,000 – 1050,000 distinct natural ligands. This value considers the number of described species and the data of the different venom analyses yielding the characterization of approx. 100–700 different venom components (*e.g.* Buthidae family: 383–632 peptides in some species of *Tityus* and *Leiurus* genera [4]; 87–144 venom components in species of the genus *Tityus*[6]; Scorpionidae family: *Pandinus cavimanus* 393 venom components [7]; *Urudacus yaschenkoi* 274 unique molecular masses [8]). Advanced methods of venom fractionation, chromatography, mass spectrometry and peptide sequencing allow the characterization of the components in scorpion venom. However, the identification of a large number of animal toxins is often also based on information obtained via transcriptome analyses. Expressed sequence tags (ESTs) from venom glands provide complementary information and often reveal not yet described components related to the biological activity of the venoms. Until now (November, 2013), 10171 scorpion nucleotide sequences were described (EST and nucleotide sequences from the databases) and only 2569 were identified as scorpion toxin or toxin-like (UniprotKB). As yet, we have discovered less than 1% of all venom components, despite the strong efforts made in this vast field to get knowledge about its considerable diversity.

*Mesobuthus gibbosus* (Brullé, 1832) is one of the most important health-threatening scorpions in Turkey. This species is considered an old species living in the Mediterranean shore of the Aegean region, including Anatolia, Greece and Aegean islands [9]. Information related to the toxin and venom compounds from *M. gibbosus* is restricted to one report [10], which describes the mRNA precursors and peptides of three alpha-potassium channel toxins (α-KTxs) [10]. No data has been reported regarding the toxin genes or genomic organization in this species.

In the present work, we described 1) the first catalogue of gene expression by transcriptome analysis of venom gland (telson) and 2) the genomic organization of the chlorotoxin genes. In order to generate the transcriptome data a cDNA library from *M. gibbosus* scorpion was constructed. The non-amplified cDNA library was randomly screened and the positive colonies carrying a DNA insert corresponding to ≥500 bp of the putative toxin transcripts were subsequently DNA sequenced and analyzed by bioinformatics tools. Our results reveal information of genes related to some cellular processes (*e.g.* NADH dehydrogenase, cytochrome, ribosomal protein, ribonuclease) and genes involved in venom gland functions (*e.g.* toxins, antimicrobial peptides, phospholipases and other putative venom peptides). We performed a comparative sequence analysis of the obtained toxin-like transcripts and the related toxin families. Three chlorotoxin-like genes from *M. gibbosus* (MegClTxs) were detected and the genomic organization of MegClTxs genes allowed us to describe a new group of the chlorotoxin family. Comparative sequence analysis with the genome of *M. martensii* and MegClTxs genes provide evidence of two ClTxs groups.

## Results and discussion

### Analysis of cDNA sequences and identification of new genes

**Figure 1:**
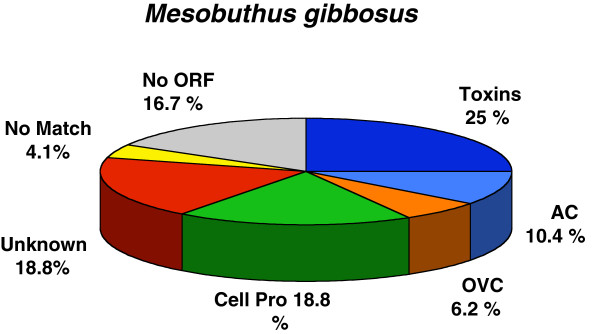
**Relative proportion of the different transcript categories in *Mesobuthus gibbosus *from the venom glands cDNA library.** Graphic colors and abbreviations of categories are as follows: dark blue colour corresponds to toxins; light blue AC, antimicrobial and cytolytic peptides; orange OVC, other venom components; green CellPro, cellular processe proteins or peptides; red unknown; yellow NoMatch and grey NoORF.

A cDNA library from *M. gibbosus* scorpion was constructed with mRNA extracted from a telson with a pair of venom glands from one specimen as previously described [10]. A random screening of 301 colonies using the non-amplified cDNA library from a pair of venom glands (one telson) was performed*.* The cDNA library clones were selected by PCR fragments, sequenced and analyzed via Phred, CAP3 and algorithms described in methods. Quality values of the DNA sequencer trace data produced by PHRED are used in the CAP sequence assembly program for overlaps between reads, removal of false overlaps and construction of contigs, generating multiple sequence alignments and consensus sequences. Results of CAP3 allow the generated contigs, singlets and quality files. A total of 201 colonies (67.7%) resulted in a sequence length corresponding to the expected size of a putative toxin or venom component transcripts (around 500 – 1000 bp). The 201 Sanger sequences were analyzed and only 177 sequences high-quality ESTs were identified as 48 Mgib sequences. Of those 48 sequences, 40 Mgib sequences (29 “singleton” and 11 “contigs”) correspond one or more ESTs. We identified 39 of these 40 Mgib sequences as putative precursor based on the corresponding ORF (the deduced amino acid sequence). To attempt the functional classification of these sequences, we compared the consensus sequences against GenBank and UniProtKB databases and we grouped the 48 Mgib sequences in different transcript categories (Figure 1). Figure 1 shows the relative proportion of categories (transcripts). We found “Toxin-like peptides” including sequences with high identity to scorpion toxin family genes (12 transcripts correspond to 25% of the total transcript sequences). “Antimicrobial and cytolytic peptides” (AC) genes (5 transcripts corresponding to 10.4% of the total transcript sequences) and "Other venom components" (OVC), described scorpion venom or secreted protein (3 transcripts correspond 6.2% of the total transcripts sequences). The category “CellPro” includes transcripts encoding for proteins involved in cellular processes (9 transcripts corresponding to 18.8%) such as enzymes, cell structure, ribosomal and other metabolism proteins. The "Unknown function" category includes ESTs with an identity of already described sequences with no functional assessment and hypothetical scorpion peptides (9 transcripts corresponding to 18.8%). The “No match” category includes ESTs that did not match with currently known sequences (2 transcripts corresponding to 4.1%) while “NoORF” describes sequence with non-identified open reading frame (8 transcripts corresponding to 16.7%). Mgib deduced amino acid sequences show a high similarity with some toxin genes from other *Mesobuthus species* and other scorpion genes (Table 1). Additional file 1: Table S1 shows the amino acid sequence deduced from the precursors of the different gene categories from *M. gibbosus*. Amino acid sequences deduced of the Mgib cDNA sequences correspond to 20 new precursor sequences that encoded to toxins-like, venom components and antimicrobial or cytolytic peptides transcripts.

**Table 1:** Annotations list of the precursor sequences deduced from the cDNA

**ID GenBank**	**Name**	**Match/% Identity**	***E-value***
**Toxins**			
KF770803	MgibC5	Potassium channel toxin alpha-KTx 10.1 [*Centruroides noxius*], 34%. 6 Cys	0.034
KF770819	MgibC10	Depressant insect toxin BmK ITa1 [*Mesobuthus martensii*], 80%. 8 Cys	1e-41
KF770809	Mgib2	Potassium channel blocker alpha-KTx 26.1 [*Mesobuthus martensii*], 70%. 6 Cys	3e-18
KF770810	Mgib3	Calcium channel toxin BmCa1, 58%. 6 Cys	7e-15
KF770808	Mgib13	Sodium channel toxin-4 [*Mesobuthus eupeus*], 85%. Partial gene, ≥8 Cys	3e-40
KF770821	Mgib23	putative potassium channel toxin Tx771 [*Buthus occitanus israelis*], 57%. 8 Cys	1e-14
KF770824	Mgib24	Potassium channel toxin BmTXK-beta-2 [*Mesobuthus martensii*], 99%. 6 Cys	2e-59
KF770827	Mgib29	Potassium channel toxin alpha-KTx 14.2 [*Mesobuthus martensii*], 91%. 6 Cys	1e-27
KF770806	Mgib49	Depressant insect toxin BmK ITa1 [*Mesobuthus martensii*], 80%. 8 Cys	5e-42
KF770800	Mgib88	venom chloride channel toxin-1 [*Mesobuthus eupeus*], 83%. 8 Cys	6e-24
KF770820	Mgib113	putative potassium channel toxin Tx771 [*Buthus occitanus israelis*], 54%. Partial gene ≥8 Cys	1e-08
KF770815	Mgib248	Sodium toxin peptide BmKTb' [*Mesobuthus martensii*], 44%. 9 Cys	5e-07
**Antimicrobial and Cytolytic**			
KF770797	MgibC1	antimicrobial peptide marcin-18 [*Mesobuthus martensii*], 81%.	4e-33
KF770807	MgibC6 (ORF1)	defensin [*Medicago truncatula*], 31%. 8 Cys	0.29
KF770812	MgibC8	Non-disulfide-bridged peptide 6.2 [*Mesobuthus martensii*], 93%.	9e-08
KF770813	MgibC9	Bradykinin-potentiating peptide NDBP6 [*Lychas mucronatus*], 85%.	2e-13
KF770816	Mgib253	Non-disulfide-bridged peptide 6.2 [*Mesobuthus martensii*], 94%.	1e-26
**Other venom components**			
KF770826	MgibC11	venom protein Txlp2 [*Hottentotta judaicus*], 79%.	1e-12
KF770814	Mgib223	venom peptide [*Hottentotta judaicus*], 28%.	5.8
KF770818	Mgib277	phospholipase A2D precursor [*Tribolium castaneum*], 49%. Partial gene.	1e-37
**CellPro**			
KF770811	MgibC7	ribonuclease R [*Coxiella burnetii* RSA 331], 33%. Partial gene	0.61
KF770822	Mgib18	zinc finger matrin-type protein 2-like [*Oryzias latipes*] Actinopterygii, 69%.	3e-89
KF770825	Mgib26	NADH dehydrogenase subunit 3 [*Mesobuthus gibbosus*], 90%.	3e-43
KF770804	Mgib36	Monogalactosyldiacylglycerol synthase, partial [Megasphaera sp. NM10], 43%	4.1
KF743063	Mgib104	cytochrome b [*Mesobuthus gibbosus*], partial, 95%	0.0
KF770799	Mgib142	Adhesive plaque matrix protein, partial [*Bos grunniens mutus*], 20%	2e-17
	Mgib263 EST	putative 40S ribosomal protein S25 [*Dolomedes mizhoanus*], 87%	6e-51
KF770817	Mgib264	Blo t profilin allergen [*Latrodectus hesperus*], 84%	2e-76
	MgibC6 (ORF2)	transposase of Tn10 [*Shigella flexneri* 2b], 100%	0.0
**Unknown (Hypothetical proteins)**			
KF770798	MgibC4	hypothetical protein 11, partial [*Urodacus yaschenkoi*], 92%.	6e-05
KF770802	Mgib1	hypothetical secreted protein [*Hottentotta judaicus*], 45%.	4e-32
KF770805	Mgib45	hypothetical secreted protein [*Hottentotta judaicus*], 77%.	3e-42
	Mgib72 EST	hypothetical protein [*Plasmodium berghei* strain ANKA], 46%.	0.070
KF770801	Mgib95	conserved hypothetical protein [Ixodes scapularis], 42%.	6e-69
	Mgib99 EST	hypothetical protein [*Capitella teleta*] Polychaeta, 25% .	2e-05
KF770823	Mgib222	hypothetical protein [*Pandinus cavimanus*], 27%.	5e-05
	Mgib267 EST	hypothetical protein [*Vibrio splendidus*]	0.53
	MgibC3	hypothetical protein 11, partial [*Urodacus yaschenkoi*], 92%.	7e-05

Match and identity of the transcripts of different gene categories are shown. *E-value* is included in the right columns.

Normally, a single-pass read of Mgib cDNA sequences includes the complete coding sequence (CDS) that corresponds with the sequence of amino acids in a peptide or protein. Mgib ESTs contain single-pass reads of the cDNA (transcript) sequence, encoding a complete precursor sequence which includes a signal peptide, mature sequence and depending of the transcript, an additional pro-peptide region. CAP3 may yield conflicting bases in the sequence generated for the contig. In order to confirm the precursors deduced from our Mgib singleton and contigs sequences and to be deposited in the GenBank database, we performed additional DNA sequencing of all obtained plasmids. Confirmed sequences, which were constructed by alignment of the group of one or more DNA sequences, was called “singleton” (named Mgib sequence) and “contig” or clusters (also named MgibClusters or MgibC) to follow the sequence analysis previously described in the transcript categories. The obtained nucleotide sequences were deposited in the GenBank database [accession numbers KF770797-KF770827, KF743063]. The annotation was based on the best match in the consulted databases (Table 1). However, some of the Mgib plasmids could not provide additional DNA sequence of high quality to complete the information of the corresponding single-pass read of cDNA sequence (see sequence Mgib EST in Table 1). These sequences were deposited in a division of the GenBank Database to the Expressed Sequence Tags (dbEST).

### Toxins-like transcripts

Scorpion venoms contain several structurally distinct families of peptidyl modulators of ion channels [11]. In accordance with the ion channel specificity, these peptides can be divided into four categories: 1) peptides of 60–70 amino acids linked by 4 disulfide bridges that modulate sodium channel activity; 2) short and long peptides of 30–76 residues with 3 or 4 disulfide bridges that block potassium channels; 3) short-chain peptides of 34–39 amino acids with four disulfide bridges and putative venom chloride channel toxin that blocks small-conductance chloride channels (ClTx) and 4) short peptides with 3 disulfide bridges that modulate ryanodine receptors (ion channels that are responsible for the release of calcium). The transcriptome analysis of *M. gibbosus* reveals a total of 12 new toxin transcripts included in the four categories of peptidyl modulators. We identified six transcripts that encode new members of the scorpion toxins specific to potassium channels belonging to the α-KTx and β-KTx families. The α-KTxs transcripts encode new toxin-like sequences of different subfamilies (by similarity with α-KTx3.x, α-KTx14.x and α-KTx26.x). Mgib24 corresponds to a new β-KTx transcript. Four different sequences encoding sodium channel toxins (NaTxs) were identified and its sequence analysis showed a match with α and β-NaTx classes. In addition, we identified a putative calcium channel toxin (Mgib3) similar to BmCa1 toxin (58% identity, *E-values* 7e-15) and a putative chloride channel toxin or chlorotoxin-like transcript (Mgib88). Our results indicate that the transcripts bear a relation to toxins from diverse scorpion genera targeting different ion channels.

### Potassium channel toxins

Scorpion toxins specific to potassium channels have been classified into families as alpha, beta, gamma (α- β- γ-KTx) [11] and kappa (κ-KTx) on the basis of the alignment of cysteines and conserved residues [12]. The α-KTx family is considered as the largest potassium channel toxin family [13]. Until now, the α-family included short-chain toxins (23–42 residues) with a total of around 150 different peptides, comprising 27 subfamilies and new peptides and precursors being continuously described (http://www.uniprot.org/docs/scorpktx). The β-KTx family, also known as long-chain potassium channel toxins (47–76 residues), has been organized into 3 groups [14], later denominated class I, II and III [15]. Peptide scorpion toxins that block the voltage-gated Shakers (K_v_1.x) channels typically consist of 30–40 residues and have a molecular weight of about 4 kDa [16]. However, β-KTxs have shown effects on some K_v_1.x channels and some members show a relation to scorpion defensins with antimicrobial activity.

### α-KTx

**Figure 2:**
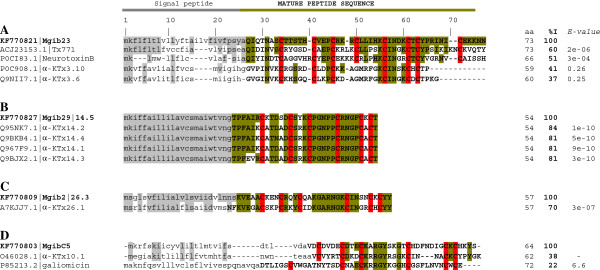
**Multiple sequence alignment of α-KTx precursors and amino acid sequences from *Mesobuthus gibbosus. *A**) Mgib23 deduced amino acid sequence and related toxins. **B**) Mgib29 precursor and all members of the subfamily α-KTx14.x. **C**) Mgib2 precursor sequence and the sole characterized member of the α-KTx26.x subfamily. **D**) MgibC5 and related precursor sequences. Signal peptides are shown in lowercase; sequences in bold and capital letters correspond to mature sequences or described toxin sequences; identical residues of mature sequences are highlighted in different colours according to the region or putative group (in more than 50% of the corresponding subfamily sequences). Number of residues, identity (% I) and *E-values* are shown on the right. Identity and *E-values* correspond to the mature sequence regions. Precursor organization is shown by cartoon of the gene (top of the figure): signal peptide is shown in grey line; pro-peptide and mature peptide is shown in green.

Five transcripts were identified that encode α-KTxs. Figure 2 shows the alignment of MegKTx and related toxins. Mgib23 encodes a toxin-like peptide precursor similar to some putative potassium channels not included in any α-KTx subfamily (more than 50% of identity of the mature sequence) and less similar to members of α-KTx12.x and α-KTx3.x families (less than 41% identity of the mature sequence) (Figure 2A). We believe Mgib23 is a member of the new α-KTx subfamily (more discussion below). Mgib29 belongs to the subfamily α-KTx14.x, which includes four members described from *M. martensii*[17,18]. α-KTx14.4 is a characterized toxin that selectively and reversibly inhibits small conductance calcium-activated potassium channels. Figure 2B shows the alignment of all precursors of the α-KTx14.x subfamily and Mgib29, with the signal peptide regions being highly conserved (100% identity). Following the nomenclature of KTxs [11], Mgib29 transcript corresponds to α-KTx14.5. Mgib2 encodes a precursor related with the α-KTx26.x. There are two members reported in this family and only α-KTx26.1 has been described (Figure 2C). The recombinant toxin α-KTx26.1 was characterized, showing an effect on K_v_1.3 channels expressed in COS7 cells [19]. According to the nomenclature, we consider that the Mgib2 transcript corresponds to the α-KTx26.x subfamily (α-KTx26.3). MgibC5 shows match and low identity with members of the α-KTx10.x subfamily. Cobatoxin- 1 and 2 are all the members of the subfamily α-KTx10.x and correspond to α-KTx10.1 and 2, respectively (from the Mexican scorpion *Centruroides noxius*). These toxins block K_v_1.x channels [20,21] (Figure 2D). The MgibC5 mature sequence showed similarity with the invertebrate defensin galiomicin (from the Lepidoptera *Galleria mellonella*). Figure 2D shown the alignment of MgibC5, α-KTx10.1 and galiomicin. The identity values (less than 38%) are too low to be considerate as a member of the same α-KTx10.x subfamily [11].

### New subfamily α-KTx 27.x

Mgib23 encodes a precursor of a toxin-like peptide similar to the putative potassium channel toxin Tx771 from *Buthus occitanus Israelis* (precursor sequence shows an identity of 57%, *E-value* 1e-14), to the putative neurotoxin B and C precursors from *Lychas mucronatus* (identity of 46% and 45%, *E-value* 2e-07) and lower identity with members of α-KTx12.x and α-KTx3.x families (Figure 2). Meg113 is a partial gene that probably corresponds to the same complete amino acid sequence from Mgib23. However, the differences in the nucleotide sequence can be taken as evidence to consider it a different transcript. The presence of 8 cysteines in the predicted mature sequence from Mgib23 and Mgib113, does not show a close relationship with subfamily α-KTx6.x members, that also possess 8 cysteines (see α-KTx6.1 sequence in the top of Figure 2). Members of the α-KTx12.x subfamily possess 8 cysteines and differ from the α-KTx6.x in the cysteines organization. Precursors of Mgib23, Tx771 and the putative neurotoxins B and C can be considered as members of the same KTx group (Additional file 2: Figure S1). Despite the lack of information related to the biological activity of members of this group, we believe that this group can be considered as a new α-KTx subfamily. According to the nomenclature for short-chain peptides, the percent of identity between α-KTxs subfamilies and database information (http://www.uniprot.org/docs/scorpktx) they correspond to α-KTx27.x [11]. The geographic distribution of scorpions is traditionally organized into two groups, namely the Old and the New World scorpions. α-KTx12.5, α-KTx12.6 and α-KTx12.7 precursors from the “Old World” (only precursors from the genus *Lychas* are described) show also differences between the “New World” α-KTx12.x members (only precursors from the genus *Tityus* are described) (Additional file 2: Figure S1). All “New World” α-KTx12.x members show a consensus sequence: WC_2_STC_4_XC_10_XC_16_XC_20_XC_31_XC_36_XC_38_YT (8 cysteines) while “Old World” members show a predicted mature sequence: QKXC_8_XC_14_XC_18_XC_29_XC_34_TC_36_YY. Perhaps, some of the “Old World” α-KTx12.x members can be reclassified since the cystine arrangement is different in the first two members of this subfamily (only 6 cysteines in the predicted mature sequence of α-KTx12.5 and α-KTx12.7). α-KTx12.6 precursor shows similar cystine arrangement to the new α-KTx27.x family members (Additional file 2: Figure S1). Mgib23 (α-KTx27.4) displays a match with α-KTx3.10 and α-KTx3.6 toxins (Figure 2 and Additional file 2: Figure S1). All described α-KTx3.x toxins correspond only to toxins belonging to scorpion species of the Buthidae family and show an effect on potassium current and specific channels [10]. However, the low identity of the Mgib23 and α-KTx3.x toxins precursors (around 30%, *E-value* 5e-05) and the discrepancy of the number of cysteines (6 cysteines in α-KTx3.x toxins) support the idea of a new KTx subfamily.

### β-KTxs

**Figure 3:**
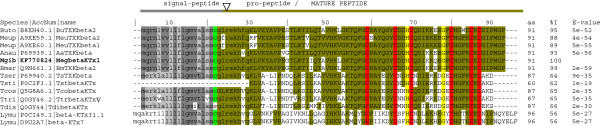
**Multiple sequence alignment of β-KTx precursors and peptides and deduced sequences from *Mesobuthus gibbosus.*** β-KTxs amino acid sequences were aligned with ClustalX. Signal peptide are shown in grey and lower case; sequence in bold and capital letters correspond to mature sequences or the N-terminal region of the described toxins; identical residues of mature sequences are highlighted in different colour according to the region or putative group; residues in yellow show identical residues in all the β-KTx class I; Number of residues, identity (% I) and *E-values* are shown on the right. Identity and *E-values* correspond to the mature sequences. Abbreviations of scorpion names, accession number of the database and toxin names are show to the left. Abbreviations of scorpion scientific names correspond to: Anau, *Androctonus australis*; Buoc, *Buthus occitanus Israelis;* Meup, *Mesobuthus eupeus;* Mgib, *Mesobuthus gibbosus;* Mema*, Mesobuthus martensii;* Tcos, *Tityus costatus;* Tser, *Tityus serrulatus*; Tsti, *Tityus stigmurus*; Ttri, *Tityus trivitatus*; Tdis, *Tityus discrepans*; Lymu, *Lychas mucronatus.* Precursor organization is shown by cartoon of the gene (top of the figure): signal peptide is shown in grey line; pro-peptide and mature peptide is shown in green; intron region is symbolized by triangles in front of the toxin TtrbetaKTx as unique example of the genomic organization of the class I [14].

β-KTxs include “chimeric” peptides with a cysteine-free N-terminal sequence and a C-terminal with a recognizable CS-αβ motif that includes 6 cysteine residues [14]. Mgib24 sequence, named Megβ-KTx1 (MegbetaKTx1 in Figure 3) is related to the group of the long-chain potassium ion channel blocker TxKbeta2 [22] and belongs to the β-KTx Class 1 [15]. All precursors of the β-KTx Class 1 reported so far belong to species of the Buthidae family (Figure 3). While the precursor of the Megβ-KTx1 shows more than 88% similarity with precursors from species belonging to “Old World” genera, there was less correspondence with species from the genus *Tityus*, which is classified among the “New World” genera. The signal peptide region has marked differences between Old World, New World, and precursors of the Chinese species *Lychas mucronatus* (Figure 3). A further distinctive and interesting feature is the conservation of consensus residues Ser-Ser-Cys, located before the pro-peptide region (position 17–19 or 19–21 in the different precursors), which suggests that it might be a conserved region involved in the post-translational processing of the precursor. The mature peptides are conserved in the putative pro-peptide regions although they are better conserved in the C-terminal. The residues H49 and G57 exhibit a high degree of conservation in all β-KTx members (to class I see residues shown in yellow, Figure 3) and additionally, residue P39 was preserved in β-KTx class I and II (complete alignment of β-KTx classes is not shown). The latter class also includes peptides poorly characterized called “orphan” peptides. Some members of the β-KTx class I have been biochemically characterized and it was discovered that they exhibit biological activity on potassium ion channels (*e.g.* TstβKTx on K_v_1.1, K_v_1.2, K_v_1.3 [23]) or potassium channels in synaptosomes (*e.g.* TsTXKβ [22]). Nevertheless, the post-translational processing and biological activity of the pro-peptides (N-terminal region) remain to be elucidated. On the other hand, genomic information of the β-KTx class I is limited to a single report concerning the genomic organization of TtrbetaKTx [GenBank: Q0GY46.2] from *Tityus trivitatus* (see intron position by symbol ∇ in Figure 3) which contains an intron that interrupts the pro-peptide region [23]. Notwithstanding the fact that currently several research groups continue to contribute valuable data related to new β-KTx members (more than 50 precursors), genetic information (8 genes) and our actual knowledge regarding the biological characterization is not sufficient to allow us to clearly understand β-KTx toxins role.

### Sodium channel toxins

**Figure 4:**
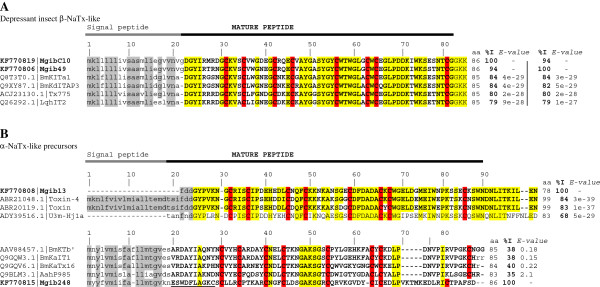
**Multiple sequence alignment of sodium channel toxins and related precursors of *M. gibbosus*.** Depressant insect β-NaTx-like (panel **A**) and α-NaTx-like precursors **(B)**. Signal peptides are shown in grey; sequences in bold and capital letters correspond to mature sequences; identical residues of mature sequence are highlighted in yellow colour and the conserved Cysteines residues are highlight in red; post-translational modification at the C-terminal is illustrated in lower case. Precursor organization is shown by cartoon of the gene (top of the figure): signal peptide is shown in grey line; pro-peptide and mature peptide is shown in black line. Identity (% I) and *E-values* are shown on the right. Identity and *E-values* correspond to the precursor sequences.

Scorpion Na^+^ channel toxins (NaTxs) modify the activity of voltage-gated sodium (Na_v_) channels. NaTxs are peptides that contain 58–76 amino acid residues in length, linked by 4 disulfide bridges (containing in general 8 cysteines) [24]. Physiological experiments have shown that these peptides modify the gating mechanisms of the sodium channels function, affecting the inactivation (α-toxins) or the activation (β-toxins) of the channel kinetics [25]. According to their effects and the binding sites on Na_v_ channels: i) α-class scorpion toxins for Na_v_ bind to channel receptor site 3 and show an effect in the inactivation mechanism of the channels [26,27]; ii) Scorpion β-class toxins modify the Na_v_ activation process by shifting activation to more negative membrane potentials after binding to site 4 [27-29]. Several NaTxs show differential effects on mammals and insects. Depending on their *in vivo* effect, the insect-specific toxins are usually classified as “excitatory” or “depressant” [26,30]. Here, four different Mgib sequences encode new NaTx’s-like peptides (Table 1, Figure 4). Mgib NaTx’s precursor sequences show a match to α- and β-NaTx: two putative depressant insect β-toxins transcripts were obtained. MgibCluster10 (MgibC10) and Mgib49 are putative depressant insect β-toxins that show 84% identity with BmKITa1 precursor [GenBank: Q8T3T0.1]. The mature sequence of MgibC10 and Mgib49 show more than 79% identity with other putative depressant insect toxins (Figure 4A). Mgib13 and Mgib248 belong to the α-NaTx class with Mgib13 being a partial clone encoding a gene very similar to precursors described from *M. eupeus* (the identity of the mature sequence corresponding to 84% with toxin-4 [GenBank: ABR21048.1] and 83% with a toxin-like precursor [GenBank: ABR20119.1]). Figure 4B shows precursors related to Mgib13, all putative toxins are reported as unknown activity. The signal peptide sequences display a strong conservation in the amino acid sequence. This phenomenon was observed within the depressant toxins family from *B. occitanus Israelis*[31]. Mgib248 shows a match to the precursor sequence of a sodium toxin peptide from *M. martensii* (44% identity, *E-value* 2e-07) (Table 1). However, this precursor shows low identity and differences in the putative mature sequence with the related toxins (Figure 4B). The predicted signal peptide shows a cleavage site between position 18 and 19 (VKN-ESW…), the mature sequence will include residues 19 to 86 (ESWDFLAGKC…) and an odd number of cysteines. Mgib248 plasmid was fully sequenced by both strands (sequence quality >30) thus excluding the possibility of an error of the number of cysteines. Previously, the odd number of cysteines has been observed in amino acid sequences deduced from scorpion cDNA (*e.g. M. martensii*[26,32] and *Androctonus crassicauda*[33]).

### Antimicrobial precursor and other venom components

**Figure 5:**
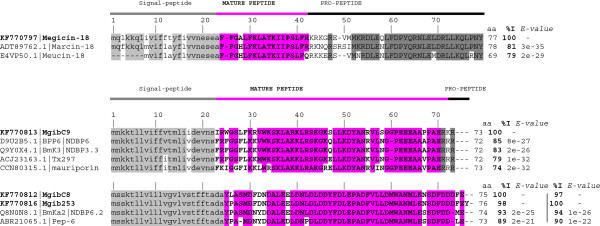
**Multiple sequence alignment of Meg antimicrobial peptides (NDBP) and related precursors.** Signal peptide are shown in lower case; sequence in bold and capital letters correspond to mature sequences; capital letters correspond to pro-peptide region. Identical residues of mature sequences are highlighted in grey or pink colour according to the precursor region shown by cartoon in the top of the figure. Number of residues, identity (% I) and *E-values* are shown on the right. Identity and *E-values* correspond to the complete precursor sequences.

Besides precursor of neurotoxins-like peptides, three Mgib transcripts correspond to venom components with other biological activities: a partial clone similar to phospholipase precursor (Mgib277) and two transcripts with high identity to scorpion cytotoxic/antimicrobial peptides (MgibC11 and Mgib223). Antimicrobial peptides can be divided into different groups according to their primary and secondary structure. One of these groups is related with the defensins widespread in different phyla and other group is called ‘cytotoxin linear peptides’. The linear peptides are mostly α-helical peptides with no cysteines called non-disulfide-bridge peptides (NDBP) [34]. Five putative transcripts were predicted to belong to the antimicrobial peptide transcripts. They correspond to the MgibC1, MgibC6 (ORF1), MgibC8, MgibC9 and Mgib253 (Figure 5). These putative antimicrobial peptides display similarity to venom components of other species (Table 1). We have identified one partial gene of a putative defensin (MgibC6, ORF1). This sequence shows low identity (31%) with the plant defensin from the barrel medic *Medicago trunculata* [NCBI: XP_003628978] and the mollusk *Hyriopsis cumingii* [GenBank: AEX88475]. Defensins conserve a cysteine-stabilized α-helix and β–sheet (αβCS) structural motif widely distributed in plants and the animal kingdom ([35,36]). Figure 5 shows the alignments of Meg-NDBPs and related precursor of NDBP families. The predicted mature sequence of Cluster1 or MegC1 (FFGALFKLATKIIPSLFR) shows similarity to precursors of the cytotoxic peptides and antimicrobial peptide members: marcin-18 and meucin-18 from *M. martensii* and *M. eupeus* respectively [GenBank: ADT89762.1 and E4VP50.1]. According to the name of homologous genes, MegC1 precursor was named Megicin-18 (Figure 5). Cluster MgibC8 and Mgib253 encode two very similar precursors related to the antimicrobial peptide from *Mesobuthus* species (identity 90-95%, *E-value* 2e-25, 2e-21 and 9e-09) and from *L. mucronatus* (identity 64% *E-value* 1e-07, [Uniprot: P0CI96.1]) of the NDBP 6 subfamily. Cluster MgibC9 is related to the bradykinin-potentiating peptides NDBP 3 subfamily. This family includes the longest peptides (40–47 residues) of the NDBP families [34]. MgibC9 precursor shows 76–89% identity (*E-value* 2e-26) with related precursors (Figure 5). Antimicrobial peptides can be viewed as an emerging class of agents because the spectra include bacteria and fungi [37]. In this respect, antimicrobial peptides are a fantastic unexplored resource for use in drug design.

### Transcripts related to cellular functions and unknown genes

We will only mention the number of transcripts obtained in the “CellPro” and “Unknown” transcript categories. Nine transcripts encoding common cellular proteins match proteins involved in diverse cell functions such as ribonuclease, NADH dehydrogenase or cytochrome b. Additional file 1: Table S1 shows the deduced amino acid sequence of CellPro and unknown transcripts. In addition, Clusters MgibC3, MgibC4 and clones Mgib1, Mgib45, Mgib72, Mgib95, Mgib99, Mgib222 and Mgib267 are similar to other scorpion hypothetical proteins with a function that remains unknown. Lastly, two “No match” ORFs were found (Additional file 1: Table S1).

### The profile of gene expression in the venom glands from Buthidae family

**Figure 6:**
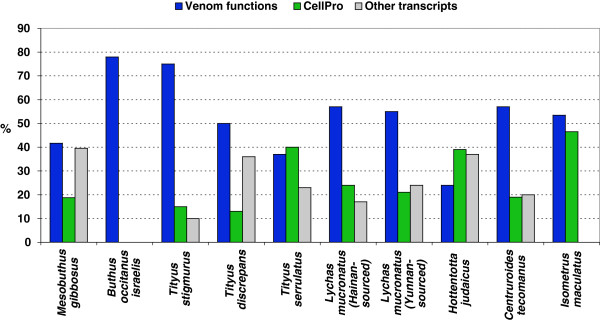
**Relative proportion of the different transcript categories in *Mesobuthus gibbosus *and comparative transcriptome analysis with other species from the Buthidae family using Sanger sequencing.** Relative proportion is shown according the original source information and integrating the sum of categories into the 3 groups: venom functions (toxins, antimicrobial, cytolytic peptides and other venom components, dark blue bars); CellPro, cellular processes proteins or peptides (green bars) and other transcripts (unknown, NoMatch and NoORF, gray bars). *Mesobuthus gibbosus* [this work], *Buthus occitanus Israelis*[31]*, Tityus stigmurus*[41], *T. discrepan*[38], *T. serrulatus*[42], *Lychas mucronatus*[39], *Hottentotta judaicus*[40], *Centruroides tecomanus*[44] and *Isometrus maculatu*s [43]*.*

In general, transcript sequences from venom glands have been used in various types of analysis: expression of different transcript categories, conservation of mature sequences, genomic organization and approaching the diversification of toxin families by gene codon usage. To the best of our knowledge, only ten reports are available describing transcriptome analyses in Buthidae family: *Tityus discrepans*[38], *Lychas mucronatus*[39], *Hottentotta judaicus*[40], *T. stigmurus*[41], *T. serrulatus*[42], *Isometrus maculatu*s [43]*Centruroides tecomanus*[44] and *B. occitanus israelis*[31] by Sanger sequencing; the transcriptome analysis from *C. noxius* performed with a pyrosequencing platform [45] and *M. martensii* sequencing by Ilumina [46]. Kozminsky-Atias *et al.*[30], used cDNA library information of the venom glands from *B. occitanus israelis* to show that the codon usage depends on the translational regulatory mechanisms, and to study the evolutionary mechanisms underlying the diversity of scorpion toxins. The genus *Mesobuthus* (synonymized as *Buthus* also) has been studied in diverse aspects related to its venom components. However, information regarding the general transcriptome analysis is still limited to *B. occitanus israelis* and *M. martensii*. Figure 6 compares the percentage of all gene categories reported of transcriptome analyses in the Buthidae scorpions by Sanger sequencing, integrating the sum of categories into 3 groups: i) transcripts of venom functions, which include toxins, antimicrobial, cytolytic peptides and venom component transcripts ii) cellular processes transcripts and iii) other transcripts including no match, no ORF and unknown functions transcripts. Comparative transcriptome analyses for the venom glands from *M. gibbosus* indicate that the abundance of toxin transcripts is high compared to the other transcriptome categories and congruent with the other Buthidae transcriptomes in similar conditions (cDNA library construction from milked scorpion). In contrast, a smaller percent of toxin transcripts was observed in the transcriptome analysis of the “resting” venom glands cDNA library from *H. judaicus* (cDNA library construction from ‘non-milked’ venom glands) and *T. serrulatus* (bars blue, Figure 6). In these cases the percentage of “CellPro” transcripts was the highest compared to other transcriptomes (bars green, Figure 6). Despite the fact that we used similar conditions for the library construction, the transcriptome analyses in the Buthidae family showed different venom compounds and differences in the percentage of gene categories. According to the original source of transcriptome information, the sum of the toxin transcript, antimicrobial, cytolytic peptide categories and other venom components corresponds to: 50% from 112 high quality ESTs in *T. discrepans*[38], to 75% from 540 high quality ESTs in *T. stigmurus*[41], 37% from 1629 high quality ESTs in *T. serrulatus,* 55% and 57% from 738 readable sequences in *L. mucronatus* (Yunnan-source) and *L. mucronatus* (Hainan-sourced) [39], 24% from 537 high-quality clones in *H. judaicus*[40], 78% from 450 clones in *B. occitanus israelis*[31]; 57% from 130 sequences in *C. tecomanus*[44], 53% from 743 readable sequences in *I. maculatu*s [43] and 41.6% using 177 high-quality ESTs in *M. gibbosus* (this work). Comparative analysis of the transcriptome analyses reported for Buthidae genus is an important tool to compare the expression of family genes in venom glands. In addition, transcriptome analyses can reveal an inter-species difference originating from different habitat, feeding behavior and other conditions (*i.e. L. mucronatus,* Figure 6). *C. noxius* transcriptome analysis was performed by a different sequencing method (pyrosequencing platform [45]). However, the three conditions used in the library construction allowed comparing the abundance and transcript level differences in each condition and in the same species [45]. At this point, the conditions used for the cDNA library construction are important elements having a repercussion on the ESTs analysis and the interpretation of the differential expression pattern(s). These conditions may also reveal intra-species (not only inter-species) differences based on the physiological state of the specimen.

In recent times, the genome of *M. martensii* revealed 32,016 protein-coding genes [46]. The authors described a total of 116 neurotoxin genes located in this genome (of which 45 were unknown), consisting of 61 NaTxs, 46 KTxs, 5 ClTxs and 4 CaTx or toxins for ryanodine receptors. In addition, Cao and colleagues [46] confirmed 109 expressed neurotoxin genes in the transcriptome analysis by next generation sequencing (NGS). The advantage and the limitations of the sequencing technology depends on factors such as the sample (venom glands) amount, focus of the study or the cost. But, all sequencing techniques allow to explore different transcriptomes from venomous species. High throughput sequencing or next generation sequencing platforms offer the possibility of generating thousands of sequences that contribute to the study of different conditions and provide a “complete” catalogue of the gene expression (*e.g.* the 72 toxin-like isogroups from *C. noxius* represent only 0.4% of the total number of assembled transcripts). In this sense, our low-throughput sequencing is far from a complete catalogue of the gene expression. However, Sanger sequencing in transcriptome is the approach often used for the screening of the cDNA libraries in the follow conditions: i) limiting sample amount (*e.g*. one or two specimens) ii) transcripts sequencing for a future characterization (*e.g.* cDNA into the vector to future recombinant protein expression) and iii) general catalogues with focus in toxin or venom component transcripts (*e.g.* selection of the estimated toxin genes by length of the PCR fragments). Our results by Sanger sequencing provided a total of 12 “toxin transcripts” (from 301 clones in the cDNA library) corresponding to 10% of the neurotoxin genes located in the *M. martensii* genome (or 11% of the expressed neurotoxins by Illumina). Rendon-Anaya *et al.*[45], identified 72 different toxin-like isogroups from *C. noxius* analysis by 454 sequencing (*e.g.* toxins, proteases, antimicrobial peptides) but only 48 toxin-like isogroups correspond to ion channel specific toxins. Our results of toxin–like transcripts to specific ion channel correspond to 25% of toxin transcripts obtained by 454 sequencing platform. The number of transcripts and information provided by the transcriptomes by Sanger sequencing is still important for the contribution to the scorpion transcripts.

### Chlorotoxin-like genes and the first scorpion genome genomic organization

**Figure 7:**
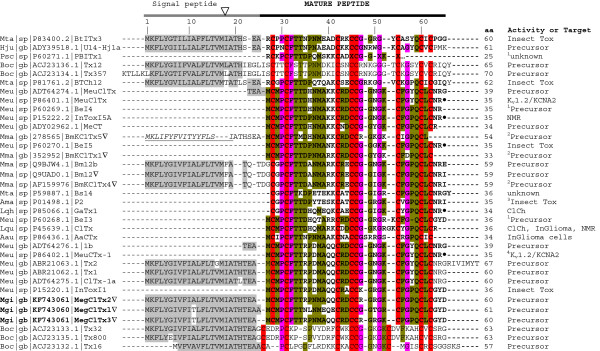
**Multiple sequence alignment of Meg toxins and related precursors of the chlorotoxin group.** Amino acid number and targets are shown on the right of the multiple alignments. Precursor denotes the deduced sequence from gene or transcript. Mature sequences are shown in bold (according to the references of the signal peptide prediction or pure peptide). Filled circle corresponds to amidated C-terminus (Arginine amide). Cysteines residues are highlighted in red. Residues highlighted in pink correspond to ≥90% identity and green to ≥50% identity. Symbol ^1^, corresponds to the deduced sequence based on amino acid analysis reported by Tytgat *et al.* 1998 [52]. Symbol ^2^, corresponds to the deduced sequence based on genome analysis reported by Cao *et al.* 2013 [46]. Symbol ^3^, corresponds to the activity reported by Rosso and Rochat 1985 [55]. Symbol ^4^, means activity reported only in UniProtKB database (data not confirmed by reference of the authors). ClCh, Chloride channel ligand; InGlioma cells, inhibition and invasion of glioma cells expressing CLCN/ClC-3 voltage-gated chloride channels. Abbreviations of scorpion scientific names correspond to: Aau, *Androctonus australis*; Ama, *Androctonus mauritanicus mauritanicus*; Boc, *Buthus occitanus Israelis*; Hju, *Hottentotta judaicos*; Lqh, *Leiurus quinquestriatus hebraeus*; Lqu, *Leiurus quinquestriatus quinquestriatus;* Meu, *Mesobuthus eupeus*; Mgi, *Mesobuthus gibbosus*; Mta, *Mesobuthus tamulus*; Psc, *Parabuthus schlechteri*.

In the second part of this work, we identified a transcript that encodes a new putative chlorotoxin (Mgib88). This sequence shows a full-length cDNA of 254 bp including the 3’-UTR [GenBank: KF770800]. Chlorotoxin is a putative venom chloride channel toxin from *Leiurus quinquestriatus hebraeus* that blocks small conductance-chloride channels and is able to bind glioma cells [47,48]. Around 30 chlorotoxin-like precursors from scorpions have been described (Figure 7), however only a few of them have been biochemically characterized (*i.e.* chlorotoxin [47] and AaCtx [49]). Prior to the characterization of chlorotoxin, the team of Prof. Grishin described several small insect toxins from *M. eupeus* (*e.g.* InTox1 [50] or InTox5 [51]). These peptides show activity on insects and show a primary structure related to the chlorotoxin precursor as homologous peptides. In addition, a partial N-terminal sequence and the deduced amino acid sequences of I3 and I4 insectotoxin-like peptides were reported [52]. The insect toxin InToxI1 [GenBank: P15220.1] is more the 90% identical to the Mgib88 deduced amino acid sequence. Furthermore, similarities between chlorotoxin and members of the α-KTXs family increased the interest in characterizing chlorotoxin-like peptides and synthetic peptides with the catalytic dyad essential to the activity on K_v_1.x channels [53,54], even though no effect on K_v_1.x channels was reported. According to the information from the GenBank/UniProtKB, the chlorotoxins MeuCTx-1 and MeuClTx (P86402.1 and P86401.1) from *M. eupeus* show an effect on K_v_1.2/KCNA2 channels. Unfortunately, the data to support this has not been published by the authors yet.

**Figure 8:**
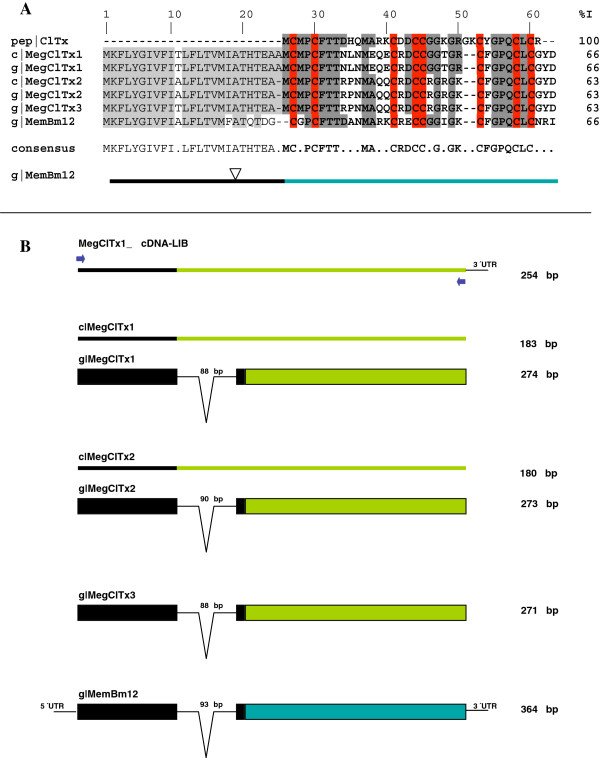
**Schematic representation of gene structures of the Meg-chlorotoxin genes from *Mesobuthus gibbosus*.** Amino acid deduced sequence and genomic organization are shown. **A**) Highlighted letters show identical amino acids between toxin sequences; putative mature sequences are in bold; Cysteines residues are highlighted in red. Abbreviations of different DNA source correspond to: c, cDNA and g, genomic DNA. The peptide is abbreviated as pep. Identity value (% I) corresponds to the mature sequences. **B**) The cartoon shows the gene topology of the three Meg-Clorotoxins-like and the chlorotoxin Bm12 from *M. martensii*[56] in the bottom of the alignment and scheme. Recently, Bm12 was named as BmKClTx3 in the genome report from *M. martensii*[46]. Large boxes are the translated sequences (signal peptide in black and mature peptide in green or blue), whereas thin lines above boxes match the deduced sequences from cDNA clones obtained. Introns (∇) and untranslated sequences (UTR) are represented as thin lines.

Mgib88 mature sequence shows a methionine as first residue similarly to other precursor sequences from *M. eupeus* and *A. autralis* (*e.g*. MeuClTx-1, AaCtx in Figure 7). Recently, the genome of *M. martensii* reveled the genomic organization of five chlorotoxin genes (BmKClTxs) [46], one of which (BmKClTx3) has been previously described and named Bm12-Chlorotoxin gene while BmKClTx4 shared its deduced amino acid sequence. Former to the report of the *M. martensii* genome, we attempted to elucidate the genomic organization of the chlorotoxin genes from *M. gibbosus* to provide evidence of the different classes of ClTxs genes by precursor and genomic organization. Signal peptide conservation of ClTxs precursors and Mgib88 were used to the design of specific screening from genomic DNA. Three ClTx genes from *M. gibbosus* were obtained from a PCR reactions, using specific primers that correspond to the precursor sequence of 183 bp (see blue arrows in Figure 8). Figure 8 shows the MegClTxs precursor sequences deduced from the cDNA and gDNA. These genes were named MegClTx1, MegClTx2 and MegClTx3 (Figure 8A and B). MegClTxs gene sequences were deposited in the GenBank database [GenBank: KF743060 to KF743062]. We performed additional experiments with cDNA to confirm the expression of MegClTxs genes. MegClTx1 and MegClTx2 are expressed in the venom glands (Figure 8A) and its deduced amino acid sequence showed an identity between the three sequences corresponding to 85% and between 63-66% with the other ClTxs (Figure 8A). All the sequences showed a small intron inserted in the signal peptide sequence (range of 88–90 bp, sequences in lower case Additional file 3: Figure S2). The genomic organization of MegClTxs genes showed similarities with the genomic organization of chlorotoxin-like Bm12 or BmKClTx3 (Figure 8B and S2). Chlorotoxin-Bm-12 gene has an intron of 93 bp [56] while the size of MegClTxs introns was in the range of 88–90 bp (Figure 8B). We compared the nucleotide sequences obtained from *M. gibbosus* and *M. martensii* genome. Despite that the genome sequences for BmKClTx1, 2 and 5 were reported as partial amino acid sequences [46], we compared sequences that correspond to the Bm12 gene, MegClTxs, the genomic sequence of the BmKClTx1 contig352952 (region sequence: 11870–11980) and BmKClTx5 contig278565 (region sequence: 1023–1268). We observed a high identity between BmKClTx1, BmKClTx5 sequences and the obtained sequence to MegClTxs (Additional file 3: Figure S2). We predicted one intron in all the sequences analyzed and a different start codon to the BmKClTx5. According with our analysis, the signal peptide to BmKClTx5 reported by Cao *et al.*[46], corresponds to the intron region predicted in Bm12 and the three MegClTxs genes (Additional file 3: Figure S2, see italic letters of the BmKClTx5 nucleotide and amino acid sequences). We could not predict a donor site to the BmKClTx5. However, the acceptor splice site is present in the same region as the other ClTxs genes in Additional file 3: Figure S2 (position 120: ctttctatag^∧^CAACTCATTC). Furthermore, the contig sequence for BmKClTx1 and BmKClTx5 include additional residues similar to MegClTxs not included in the deduced amino acid sequence reported by Cao *et al.*[46] (Additional file 3: Figure S2, see residues in gray and the stop codon of the BmKClTx1 and BmKClTx5 sequences). Our results thus provide information to complete and generate a second group in the chlorotoxin family from genus *Mesobuthus*.

## Conclusions

This report revels part of the diversity of genes expressed in the venom glands from *M. gibbosus*. We identified several transcripts of toxic relevance as evidenced by orthologous genes. Furthermore, ribosomal and housekeeping transcripts were obtained. The transcriptome analysis revealed new putative peptides and may help to identify putative post-translational modifications in the deduced amino acid precursor sequences of the transcripts. In addition, for the chlorotoxin family genes, we described the genomic organization of three new genes and confirmed the corresponding expressed sequences for two of them. This information may contribute to the classification of chlorotoxin genes into two groups for the genus *Mesobuthus*. This transcriptome contribution can be useful for further studies and to help discovery new gene families, toxins and other venom components.

## Methods

### Biological materials and cDNA library construction

A cDNA library of *M. gibbosus* venom glands was generated using the conditions previously described [10]. A random sequencing strategy was used to screen the cDNA library*.* In order to select the positive colonies, random screening using blue/white colony selection (by non-functional β–galactosidase activity, consequence of the *LacZ* gene disruption by Mgib sequences or transcripts) and colony polymerase chain reaction (colony PCR) was performed. The PCR fragments selected correspond to the expected length of toxin and venom components transcripts (around 500–1000 bp). The selection of positive clones by colony PCR was done using forward and reverse primer screening (sites flanking pSMART21F inserts). The plasmid DNA of selected colonies was obtained by mini-prep kit preparations (Roche) and sequenced by Sanger method from both ends by GATC Biotech sequencing service (Germany).

### Genomic organization

Genomic DNA was obtained from the legs and tail of two specimens of *M. gibbosus* following the protocol described by Rodriguez de la Vega and colleagues [57]. To obtain ClTxs genes from genomic DNA, we designed specific primers based in the information obtained by conserved signal peptide sequences (Figure 7) and the DNA sequence of Mgib88, the putative chlorotoxin from *M. gibbosus* (GeneBank: KF770800): 5’- ATG AAG TTC CTC TAT GGA ATC GTT TTC −3’ and 5’- TCA GTC ATA GCC ACA CAG ACA TTG TGG −3’. PCR products were amplified using the conditions described Diego-Garcia *et al.*[23]. High Fidelity Taq polymerase (Roche) was used in the PCR reactions. PCR products were cloned in a pGEM vector (Promega) and sequenced by the Sanger method.

### Bioinformatic analysis of DNA sequences

DNA sequences were analyzed by electropherogram quality analysis via the PHRED web service [58] and assembled in clusters using the CAP3 program [59]. Sequences were processed as follows: unique sequences are considered singletons or singlets. An assembly of contiguous sequences is considered a contig. Additionally, all the plasmids included in the singletons and contigs were reverse strand sequenced to confirm the final deposited sequence in the GenBank database. Confirmed sequences were called “singleton” (named Mgib sequence) and “contig” or cluster (also named MgibCluster or MgibC). Each sequence was searched against the GenBank database with algorithms BLASTX and Protein BLAST to identify homologous sequences for comparison [60]. All DNA sequences were manually inspected with DNA Strider 1.4f6 to identify open reading frames (ORF), 4Peaks 1.7.2 tools to confirm the nucleotide sequence and the multiple sequence alignment program and Clustalx 1.83.1 or ClustalW2 (http://www.ebi.ac.uk/Tools/msa/clustalw2). SignalP 4.0 [61] and ProP 1.0 [62] servers were used for the prediction of the presence and location of signal peptide and pro-peptide cleavage sites respectively. All Mgib sequences that encode for toxin-like, antimicrobial and venom compounds were fully sequenced by reverse and forward DNA strand to be submitted to GenBank [KF770797-KF770827, KF743063]. Singletons were submitted to EST database from GenBank. Splice sites predictions to identify the exon-intron regions were obtained by using NetGene2 v2.4 [63].

### Ethical statement

The authors and co-authors of this paper have acted ethically in conducting the described research, having careful analysis of the data to avoid errors. Authors declare that the described work has not been published previously. All authors approve this manuscript.

## Abbreviations

Mgib: Nucleotide or amino acid sequence deduced from cDNA; Meg: Toxin-like or precursor deduced of the cDNA sequence from Mesobuthus gibbosus; 5’-UTR: 5’, Untranslational region; 3’-UTR: 3’, Untranslational region; EST: Expressed sequence tag; MALDI-TOF MS: Matrix-assisted laser desorption ionization time-of-flight mass spectrometry; MS: Mass spectrometry; NaTx: Sodium channel toxin; NDBP: Non-disulfide-bridge peptides; TFA: Trifluoroacetic acid; Nav: Voltage-gated sodium channels; KTx: Potassium channel toxin; Kv: Voltage-gated potassium channels.

## Competing interests

The authors declare that they have no competing interests.

## Authors’ contributions

EDG conceived and designed the study, carried out the molecular studies, performed the analysis and interpretation of the data, drafted and revised the manuscript. FC carried out the sample preparation from the telson. JT conceived the study, participated in the coordination and helped to finalize the manuscript. All authors read and approved the final manuscript.

## Supplementary Material

Additional file 1: Table S1 Predicted amino acid sequences of the putative venom compounds and other peptides from the venom glands cDNA library of *Mesobuthus gibbosus*. Complete and partial genes of different categories are shown, identity and *E-value* are included in the right column. Putative mature sequences are in bold; putative pro-peptides are underlined; an asterisk indicates a stop codon; 3 points in the start or at the end of the sequences as a reference to the partial precursor sequence that is located in the C-terminal or N-terminal. Symbol ≥ means that the total number of cysteines could be higher in the complete precursor sequence. Click here for file

Additional file 2: Figure S1 Multiple sequence alignment of Mgib23 (alpha-KTx27.4) and related precursors or toxins. The alignment shows mature sequences in bold; highlighted letters show identical nucleotides in gray (signal peptide) and mature sequences (green). Abbreviations of scorpion scientific names correspond to: ANUPH, *Anuroctonus phaiodactylus*; BUTOS, *Buthus occitanus israelis* (*Mesobuthus occitanus israelis*); HADGE, Hadrurus gertschi; HEMLE, *Hemiscorpius lepturus*; HETSP, *Heterometrus spinifer*; HOTJU, *Hottentotta judaicus*; LYCMC, *Lychas mucronatus*; MESGI, *Mesobuthus gibbosus*; MESMA, *Mesobuthus martensii*; OPICA, *Opistophthalmus carinatus*; OPIMA, *Opisthacanthus madagascariensis*; OPICY, *Opisthacanthus cayaporum*; PANIM, *Pandinus imperator*; SCOMA, *Scorpio maurus palmatus*; TITCO, *Tityus costatus*; TITSE, *Tityus serrulatus*; TITST, *Tityus stigmurus*; TITTR, *Tityus trivittatus*. Color abbreviations correspond to scorpion families: blue, Scorpionidae; red, Hemiscorpiidae; green Buthidae; orange, Iuridae; brown, Liochelidae. Amino acid number and *E-value* are included in the right columns. Abbreviation nd correspond to undetermined. Click here for file

Additional file 3: Figure S2 Nucleotide sequences and deduced amino acid sequences of the Meg-chlorotoxins-like from *M. gibbosus*. Nucleotide sequences and the corresponding amino acid sequence deduced from gDNA. In the Genomic DNA sequences (gMeg) the exons are written in capital letters; the introns sequences are in lower and; highlighted letters show identical nucleotides in gray. Amino acid sequences (aMeg) show the predicted signal peptides underline; putative mature sequences are in bold; cysteines residues are highlighted in red. The alignment includes Bm12 gene from *Mesobuthus martensii*[34] and the amino acid sequence of the Chlorotoxin from *Leiurus quinquestriatus quinquestriatus*[32]. Most of the eukaryotic proteins-coding genes are interrupted by introns that are removed at the donor and acceptor splice sites such that the adjacent exons are spliced. Introns occur in three phases that are defined as the position of the intron within or between codons: intron of phases 0, 1 and 2 are located respectively, between two codons, after the first position in a codon, and after the second position [64]. MegClTxs introns have a consensus splice sites of gt at the 5’-end and ag at the 3’-end. Donor splice sites to exon-intron were conserved to the three MegClTxs genes (5’GTAATGATCG^∧^gtaagtgatt3’), showed phase 0 to MegClTx1 and MegClTx 3, while MegClTx2 was phase 2. Acceptor splice sites intron-exon was conserved to the three genes also (5’ccttttatag^∧^CAACTCATAC3’), all the genes showed phase 2. Bm12 gene has the same sequence to the donor splice sites than MegClTx2 and showed phase 2. However, it shows a different sequence to the acceptor splice sites phase 1 (atttatgtag^∧^CAACTCAAAC). Click here for file
