# Supplementary material for: The Mediterranean scorpion Mesobuthus gibbosus (Scorpiones, Buthidae): transcriptome analysis and organization of the genome encoding chlorotoxin-like peptides
Source: BMC Genomics. 2014 Apr 21;15:295. doi: 10.1186/1471-2164-15-295 (PMC4234519; doi:10.1186/1471-2164-15-295)
Supplement: Additional file 1: Table S1 — Predicted amino acid sequences of the putative venom compounds and other peptides from the venom glands cDNA library of Mesobuthus gibbosus. Complete and partial genes of different categories are shown, identity and E-value are included in the right column. Putative mature sequences are in bold; putative pro-peptides are underlined; an asterisk indicates a stop codon; 3 points in the start or at the end of the sequences as a reference to the partial precursor sequence that is located in the C-terminal or N-terminal. Symbol ≥ means that the total number of cysteines could be higher in the complete precursor sequence. [file 1471-2164-15-295-S1.doc]

**Table S1.** **Predicted amino acid sequences of the putative venom compounds and other peptides from the venom glands cDNA library of *Mesobuthus gibbosus*.**

| **Name** | Sequence | Match / % Identity | E-value |
| --- | --- | --- | --- |
|  | Toxins-like |  |  |
| MgibC5 | MKRFSKIICYVLILTLMTVIFSDTLVDAVDCDVDECDTECKARGYSKGTCHDFNDIGCKCHKYS* | Potassium channel toxin alpha-KTx 10.1 [Centruroides noxius], 34%. 6 Cys | 0.034 |
| MgibC10 | MKLLLLLIVSASMLIEGVVNVGDGYIRMRDGCKVSCVWGDEGCRQECVAYGASYGYCWTWGLGCWCEGLPDDKIWKSESNTCGGKK* | Depressant insect toxin BmK ITa1 [Mesobuthus martensii], 80%. 8 Cys | 1e-41 |
| Mgib2 | MSGLSVFILIALVLSVIIDVLNNSKVEAACKENCRQYCQAKGARNGKCINSNCKCYY* | Potassium channel blocker alpha-KTx 26.1 [Mesobuthus martensii], 70%. 6 Cys | 3e-18 |
| Mgib3 | MNRFFILLLLIVILSHAKAEDESYRGNCPSLKKPCNSDRDCCPYGEKCLSAGAGYYCKPDPGP* | Calcium channel toxin BmCa1, 58%. 6 Cys | 7e-15 |
| Mgib13 | …FDDGYPVKNGCRISCIPDEHEDLCNQFCKKNKANSGECDFDADACKCWGELDGMEIWNPKSSECKSWNDNLITKILEN* | Sodium channel toxin-4 [Mesobuthus eupeus], 85%. Partial gene, ≥8 Cys | 3e-40 |
| Mgib23 | MKFLFLTLVLLYFTAILVFIVFPSYAQIQTNASCTTSTHCVEPCRKRCLLIHKCINDKCTCYPRINICEKKNN* | putative potassium channel toxin Tx771 [Buthus occitanus israelis], 57%. 8 Cys | 1e-14 |
| Mgib24 | MQRNLVVLLFLGMVALSSCGLREKHFQKLVKYAVPEGTLRTIIQTAVHKLGKTQFGCPAYQGYCDDHCQDIKKQEGFCHGFKCKCGIPMGF* | Potassium channel toxin BmTXK-beta-2 [Mesobuthus martensii], 99%. 6 Cys | 2e-59 |
| Mgib29 | MKIFFAILLILAVCSMAIWTVNGTPFAIRCKTDSDCSYKCPGNPPCRNGFCKCT* | Potassium channel toxin alpha-KTx 14.2 [Mesobuthus martensii], 91%. 6 Cys | 1e-27 |
| Mgib49 | MKLLLLLIVSASMLIEGVVNVGDGYIRTRNGCKVSCVWGNEGCRQECVAYGASYGYCWTWGLGCWCEGLPDDKIWKSSTNTCGGKK* | Depressant insect toxin BmK ITa1 [Mesobuthus martensii], 80%. 8 Cys | 5e-42 |
| Mgib88 | MKFLYGIVFITLFLTVMIATHTEAAMCMPCFTTNLNMEQECRDCCGGTGRCFGPQCLCGYD* | venom chloride channel toxin-1 [Mesobuthus eupeus], 83%. 8 Cys | 6e-24 |
| Mgib113 | ...FIVFPSYAQIQTNASCTTSTHCVEPCRKRCLLIHKCINDKCTCYPRINICEKKNN* | putative potassium channel toxin Tx771 [Buthus occitanus israelis], 54%. Partial gene ≥8 Cys | 1e-08 |
| Mgib248 | MYYFVMIIFALLMTGVKNESWDFLAGKCSCLLRCPTKARCNGFCLDSGAKSGRCQRVKGVDYCICEDLPVKTMKEDLRICTPAFSD* | Sodium toxin peptide BmKTb' [Mesobuthus martensii], 44%. 8 Cys | 5e-07 |
|  | Antimicrobial and Cytolytic |  |  |
| MgibC1 | MQLKKQLIVIFFTYFIVVNESEAFFGALFKLATKIIPSLFRKRKGREVMMKRDLEQLFDPYQRNLELDRLLKQLPNY* | antimicrobial peptide marcin-18 [Mesobuthus martensii], 81%. | 4e-33 |
| MgibC6 (ORF1) | …WDTCWHPLYKMKVVFIIMLLVLACCINENLVDARCPFSSSRCVLHCRDNGFGSGKCKWFKCRCLKKKK* | defensin [Medicago truncatula], 31%. Partial gene 8 Cys | 0.29 |
| MgibC8 | MSSKTLLVLLLVGVLVSTFFTADAYLASMDFDNDALEELDNLDLDDYFDLEPADFVLLDMWANMLENSDFDDDFE* | Non-disulfide-bridged peptide 6.2 [Mesobuthus martensii], 93%. | 9e-08 |
| MgibC9 | MNKKTLLVIFFVTMLIIDEVNSIRWGSLFKRVWKSKLARKLRSKGKSLLKDYANRVLSGGPEEEAAPPAERKR* | Bradykinin-potentiating peptide NDBP6 [Lychas mucronatus], 85%. | 2e-13 |
| Mgib253 | MSSKTLLVLLLVGVLVSTFFTADAYPASMDFDNDALEELDNLDLDDYFDLEPADFVLLDMWANMLENSDFDDDFEY* | Non-disulfide-bridged peptide 6.2 [Mesobuthus martensii], 94%. | 1e-26 |
|  | Other venom components |  |  |
| MgibC11 | MTLTNGLSLRTIFITLLLLLPPHLLATTASITKIQTRNRTRKVRGFILLSEAAENRD* | venom protein Txlp2 [Hottentotta judaicus], 79%. | 1e-12 |
| Mgib223 | MKRFLVFSILFQTVFCMKTSDQVGIITYQGVPRRERKCILGPWIHLEDGSVFHDSDRCEIKTCHITAQKAYLEVQSCRYKVNCERQILEPYFPHCCPTSPKCT* | venom peptide [Hottentotta judaicus], 28%. 8 cys | 5.8 |
| Mgib277 | ...WCGAGSSAKHEDELGRFNDTDSCCRHHDHCHDNIKGKETKYGLKNKDSTTMSHCDCDEEFYACLKTVDSIISNNVGNMFFNFLQKKCFREDYPIKRCVKKSLIRRRCKKYELDRTKPKIWQIFDPKEY* | phospholipase A2D precursor [Tribolium castaneum], 49%. Partial gene. ≥10 Cys | 1e-37 |
|  | Cellular Proteins |  |  |
| MgibC6 (ORF2) | MCELDILHDSLYQFCPELHLKRLNSLTLACHALLDCKTLTLTELGRNLPTKARTKHNIKRIDRLLGNRHLHKERLAVYRWHASFICSGNTMPIVLVDWSDIREQKRLMVLRASVALHGRSVTLYEKAFPLSEQCSKKAHDQFLADLASILPSNTTPLIVSDAGFKVPWYKSVEKLGWYWLSRVRGKVQYADLGAENWKPISNLHDMSSSHSKTLGYKRLTKSNPISCQILLYKSRSKGRKNQRSTRTHCHHPSPKIYSASAKEPWILATNLPVEIRTPKQLVNIYSKRMQIEETFRDLKSPAYGLGLRHSRTSSSERFDIMLLIALMLQLTCWLAGVHAQKQGWDKHFQANTVRNRNVLSTVRLGMEVLRHSGYTITREDSLVAATLLTQNLFTHGYVLGKL* | transposase of Tn10 [Shigella flexneri 2b], 100% |  |
| MgibC7 | MKLILLLIVMGILAISKCSVGNTLCELSRNQKIVLLECLESYLTEEQKASEYQFYQCLGYDSVIDYYEEICGLSEKEQEELHLSYIKCHNEMTPYSGSATDEDARNCLNNAIESEQ* | ribonuclease R [Coxiella burnetii RSA 331], 33%. Partial gene | 0.61 |
| Mgib18 | MATTSKPEDHRKKWDRDEFERLAHERILEEYELEKKAKDKQPPIRREYLKPRDYRVDLESKLGKTTVITKTTPASQAGGYYCNVCDCVVKDSINFLDHINGKKHQRNLGMSMRVERSTLEQVKKRFEMNRKKLDEKKKEYDFEQRMQELRDEEEKLKEYRRERRKERKRKCQDSRDDDIDPEMAAIMGFSEFGSSKK* | zinc finger matrin-type protein 2-like [Oryzias latipes] Actinopterygii, 69%. | 3e-89 |
| Mgib26 | …VLLLVLVFRILLRSIILFVGGVISKKLEVEKEKGSVYECGFESRKRARVPFSLQFFMVGVIFLIFDVEIVLIMPVPLEAWLRGGEVLLFFFFVFLLLFGLFFE* | NADH dehydrogenase subunit 3 [Mesobuthus gibbosus], 90%. | 3e-43 |
| Mgib36 | …MLISKIIKRIDWFPSVFKYFYRSERGYRKGLRRKPISVISENKNEDAKQIFKRDIMENLKQE... | Monogalactosyldiacylglycerol synthase, partial [Megasphaera sp. NM10], 43% | 4.1 |
| Mgib104 | GGILRNLHLNGARIFFVCLYFHIGRGIYFGSFKFYFTwITGVVIFLLTIITAFLGYVLPwGQMSFwGATVITNLVSAVPYIGSEVVQWLwGGFSVDNPTLTRFFSFHFLLPFVILGRVIIHLIFLHERGSRNPLGVKRDFDKIPFHPYFSLKDLLGALFLIIFLIFISLLFPNFLSDPENCIPANPLVTPVHIQPEwYFLFAYAILRSIPNKLGGVIALIMSVLILILLSFSTSKSRAFSFRIGSRSLFwILVNIFILLTwIGARPVEFPYIMLGQGLS | cytochrome b [Mesobuthus gibbosus], partial, 95% |  |
| Mgib142 | MGIVTVHKKKSNLKMNKGTMFLSFILILLIVDGIKSYNIEREEELEERLLDDDLKEELEENRNELRQKRDRYLPTVQKRERYLPTVQKRERYLPTVQKRERYLPTVQKRERYLPTVQKRERYLPTVQKRERYLPTVQKRERYLPTVQKRERYLPTR* | Adhesive plaque matrix protein, partial [Bos grunniens mutus], 20% | 2e-17 |
| Mgib263 EST | MPPKQDQKKKDTKGTTKKKEGASSGGKAKKKKWSKGKVRDKLNNLVLFDKATYEKLLKEVPSYKLITPSVVSERLKVRGSLARRALEELRQKGLIKQVVKHHSQIIYTRTTKADDST* | putative 40S ribosomal protein S25 [Dolomedes mizhoanus], 87% | 6e-51 |
| Mgib264 | MSWQAYVDNQICAQVSCRLAVIAGLQDGAIWAKFEKDMPKPVTQQELKLIADTMRTNPNSFTESGIRLGEDKYYCLHAENSLLRGRKGSSALIVVATNTCLLVAATTDGFPPGQLNAVVEKLGDYLRSNNY* | Blo t profilin allergen [Latrodectus hesperus], 84% | 2e-76 |
|  | **Unkown (hypothetical)** |  |  |
| MgibC3 | GVCSTFKIFHDLSSNRCKPGWFLS | hypothetical protein 11, partial [Urodacus yaschenkoi], 92%. | 7e-05 |
| MgibC4 | GVCSTFKIFHDLSSNRCKPGWFLS | hypothetical protein 11, partial [Urodacus yaschenkoi], 92%. | 6e-05 |
| Mgib1 | MKNLAIITLSLISLSTSFSLFPSLSLPSVPLYPSSLIPLFMKDPVKEITKIYCVLKNVNIARSCARENNQGKISDFFRKCMQKITSLKTLDEMRDFYCNKMNLEETFKAKACLDPGIMNMVLTDATFVPTILNCLFKAKNSEK* | hypothetical secreted protein [Hottentotta judaicus], 45%. 6 Cys | 4e-32 |
| Mgib45 | MKPVQSVVLFLLLLALHASLSARIARQAEDLDDENNEVPQDEDSALDPDEDSEEDDDDGFSFRFSWNPFQNFPDIFKQMRDNMNWIYSNLFNETSNLPEVYKNVSSEIVTIGGSKFNVSKTIIKKADNNSQVVISSVSMDQVNKKKK* | hypothetical secreted protein [Hottentotta judaicus], 77%. | 3e-42 |
| Mgib72 EST | …SSSLPEAVLSVFRLLPLSSISLTLRFTVILRTSGITSHKTCYPDVPKMPGTIKKTSLLALCP* | hypothetical protein [Plasmodium berghei strain ANKA], 46%. Partial gene | 0.070 |
| Mgib95 | MMESLSCVGLSYALIEYIVNSDLSLFYPQLKSCLFLASCEECILDVESYCSFSPPNKSVAVKEHVLAACKIYVEGREGVIILDPGYHVGIPIVAMKDGLYPHKGWFVQSETPKSKKEYCYQLKGDYVHWTVRETRNGKEETWCNLIYIKKKFLSYISVSEKRNLVFNFRTLVARDEKQPVGGFYCNLEGDEKFTLFFKDYLGKRIEIKIPFGYFKGSRNNNEYESAIRKCAVQIKTNSALLVGMMTQLVEAYYDKEFMLPVNEINREIDED* | conserved hypothetical protein [Ixodes scapularis], 42%. | 6e-69 |
| Mgib99 EST | …EEICHMLTKYIKLRKLPELFSKLFASLENNSTEKNLIYPAEFLQDLSSQISYLPAGQILEIWKICLVNGSKLLNDIKKSKNEISIKCLNMIAEILRIFILHFKIDRDICLSSKKQTELENITVFIKKFMNICIKKNIQADLGYSILSLCYVWGELHLSMKHYNRFYQFKIEFTKEDKGLFDLNAIHYYFTSQEWTDLLNNFENIKENKIQY… | hypothetical protein CAPTEDRAFT_188127 [Capitella teleta] Polychaeta, 25% . Partial gene | 2e-05 |
| Mgib222 | MKISIALVLGMIACSFVCINGGDFDQYCSIPSNIRELVWNCIYIYLPQSEKTVFSQMSQCFGVSTFDKVINQFCGLSDDEQETVALQHEACLSEIDFDNFHGPTVSQYQTQSCIQRRLG* | hypothetical protein [Pandinus cavimanus], 27%. | 5e-05 |
| Mgib267 EST | …VPFFFSGIFFFEHAQFLHYQVFKKMFHSHISIMNIFW* | hypothetical protein [Vibrio splendidus]. Partial gene | 0.53 |
|  | No Match |  |  |
| Mgib55 EST | MVISYLYFLFEIFLEGSVKRGGGVFFGGVFENYVMGGREPLFAGKVALCCFKKNFFSVF* |  |  |
| Mgib16 EST | MGGVSPLLAPPVFPFGVPVFFFGGIWGGKNFPPGNPFAPVFPPVPPPRGGSWGSPPGGPTRGPGGGPSFPRGAKFCGSLRTPPKNFF* |  |  |
