# Supplementary material for: The Mediterranean scorpion Mesobuthus gibbosus (Scorpiones, Buthidae): transcriptome analysis and organization of the genome encoding chlorotoxin-like peptides
Source: BMC Genomics. 2014 Apr 21;15:295. doi: 10.1186/1471-2164-15-295 (PMC4234519; doi:10.1186/1471-2164-15-295)
Supplement: Additional file 2: Figure S1 — Multiple sequence alignment of Mgib23 (alpha-KTx27.4) and related precursors or toxins. The alignment shows mature sequences in bold; highlighted letters show identical nucleotides in gray (signal peptide) and mature sequences (green). Abbreviations of scorpion scientific names correspond to: ANUPH, Anuroctonus phaiodactylus; BUTOS, Buthus occitanus israelis (Mesobuthus occitanus israelis); HADGE, Hadrurus gertschi; HEMLE, Hemiscorpius lepturus; HETSP, Heterometrus spinifer; HOTJU, Hottentotta judaicus; LYCMC, Lychas mucronatus; MESGI, Mesobuthus gibbosus; MESMA, Mesobuthus martensii; OPICA, Opistophthalmus carinatus; OPIMA, Opisthacanthus madagascariensis; OPICY, Opisthacanthus cayaporum; PANIM, Pandinus imperator; SCOMA, Scorpio maurus palmatus; TITCO, Tityus costatus; TITSE, Tityus serrulatus; TITST, Tityus stigmurus; TITTR, Tityus trivittatus. Color abbreviations correspond to scorpion families: blue, Scorpionidae; red, Hemiscorpiidae; green Buthidae; orange, Iuridae; brown, Liochelidae. Amino acid number and E-value are included in the right columns. Abbreviation nd correspond to undetermined. [file 1471-2164-15-295-S2.doc]

# Figure S1

1 10 20 30 40 50 60 70

| | | | | | | | aa **%I**

sp|P80719|KTx6.2_**SCOMA**  ------------------------------**---VSCTGSKD-CYAPCRKQTGCPNAKCINKSCKCYG-C-**---------- 34 33

sp|P58490|KTx6.5_**PANIM**  ------------------------------**DEAIRCTGTKD-CYIPCRYITGCFNSRCINKSCKCYG-CT**---------- 38 25

sp|P58498|KTx6.4_**PANIM**  ------------------------------**IEAIRCGGSRD-CYRPCQKRTGCPNAKCINKTCKCYG-CS**---------- 38 25

sp|P84094|KTx6.13_**HETSP** ------------------------------**---IRCSGSRD-CYSPCMKQTGCPNAKCINKSCKCYG-C-**---------- 34 27

sp|Q6XLL6|KTx6.9_**OPICA** --mnakfillllvvttttllp-----dakg**AEIIRCSGTRE-CYAPCQKLTGCLNAKCMNKACKCYG-CV-**--------- 61 21

sp|Q6XLL5|KTx6.10_**OPICA** --mnakfilllvltt-mmllp-----dtkg**AEVIRCSGSKQ-CYGPCKQQTGCTNSKCMNKVCKCYG-CG-**--------- 60 23

sp|P86116|KTx6.17_**OPICY** ------------------------------**---IRCQGSNQ-CYGHCREKTGCMNGKCINRVCKCYG-C--**--------- 34 25

sp|Q10726|KTx6.1_**PANIM** ------------------------------**--LVKCRGTSD-CGRPCQQQTGCPNSKCINRMCKCYG-C--**--------- 35 23

sp|P85528|KTx6.15_**HEMLE** ------------------------------**---IKCTLSKD-CYSPCKKETGCPRAKCINRNCKCYG-CS-**--------- 35 27

sp|Q6XLL9|KTx6.6_**OPICA**  --mnakfillllvvattmllp-----dtqg**AEVIKCRTPKD-CAGPCRKQTGCPHGKCMNRTCRCNR-CG-**--------- 61 25

sp|Q6XLL8|KTx6.7_**OPICA**  --mnakfillllvvtttmllp-----dtqg**AEVIKCRTPKD-CADPCRKQTGCPHGKCMNRTCRCNR-CG-**--------- 61 25

sp|Q6XLL7|KTx6.8_**OPICA**  --mnakfillllvvtttillp-----dtqg**AEVIKCRTPKD-CADPCRKQTGCPHAKCMNKTCRCHR-CG-**--------- 61 25

sp|P59867|KTx6.3_**HETSP**  ------------------------------**---ASCRTPKD-CADPCRKETGCPYGKCMNRKCKCNR-C--**--------- 34 31

sp|P0C908.1|KTx3.10_**BUTOS**  --mkvffa-vlialfvcsmv---igihg**GVPINVKCRGSRD-CLDPCKKAGMRFG-KCINSKCHCTP--**----------- 59 41

gb|ACJ23138.1|Tx260_**BUTOC**  --mkvffa-vlitlfvcsmi---igihg**GVPINVKCRGSRD-CLDPCKKAGMRFG-KCINSKCHCTP-W**----------- 60 41

gb|AAD47376.1|KTx_**MESMA** --mkvffa-vlitlfissmi---igihg**-VGINVKCKHSGQ-CLKPCKDAGMRFG-KCINGKCDCTP-K**G---------- 60 38

sp|Q9NII7.1|KTx3.6_**MESMA** --mkvffa-vlitlficsmi---igihg**-VGINVKCKHSGQ-CLKPCKDAGMRFG-KCINGKCDCTP-K**G---------- 60 37

gb|ADY39614.1|U8-Hj3a_**HOTJU** --MKILSV-LLLAFIICSIV---YWSEAEITDES-CEFSIH-CLLVCRKKFEYLQVKCVSGKCHCYP-D----------- 60 46

sp|P0CI47.1|KTx12.6_**LYCMC** MKMKIFIITIVIALFITSIV------EAQNKLDVKCVRLET-CREPCKKQLCLLPMKCMNGKCVCSP-SRKI--C----- 65 44

gb|KF770821|**Mgib23_MESGI**|**KTx27.4** --mkflfltlvllyftailvfivfpsya**QIQTNASCTTSTH-CVEPCRK-RCLLIHKCINDKCTCYP-RINI--CEKKNN** 73 100

gb|ACJ23153.1Tx771_**BUTOS**|**KTx27.1** --mkflfltlfvccfiavlv---ipsea**QIDINVSCRYGSD-CAEPCKRLKCLLPSKCINGKCTCYP-SIKIKNCKVQTY** 73 60

sp|P0CI83.1|NeuTxB**_LYCMC**|**KTx27.2** --mklmwl-lflcvlafsia--------**QIYINDTCAGGVHRCYEPCEKKKCRLPHKCINGRCTCYV-GRNV--CAISSH** 66 51

sp|P0CI84.1|NeuTxC**_LYCMC**|**KTx27.3** --mklmwl-lflcvlafsia--------**QIYISDPCAGGVHRCYEPCEKKKCRLPHKCINGRCTCYV-GRNV--CAISSH** 66 49

sp|P0CH12.1|KTx12.5_**LYCMC** --MNKLPILIFMLLVCSMFIS----SDCQKHTDIKCSSSSS-CYEPCRGVTGRAHGKCMNGRCTCYY------------- 60 46

sp|P0CI48|KTx12.7_**LYCMC** --MSNMPVLIITLLLFSMYIS----TAAQKPTEIKCRYPAD-CHIMCRKVTGRAEGKCMNGKCTCYY------------- 60 29

sp|P59936|KTx12.1_**TITSE** --------------------------**WCSTCLDLACGASRE-CYDPCFKAFGRAHGKCMNNKCRCYT**------------- 40 29

sp|P0C168|KTx12.2_**TITTR** --------------------------**WCSTCLDLACGASRE-CYDPCFKAFGRAHGKCMNNKCRCYT**------------- 40 29

sp|P0C8L1|KTx12.4_**TITST** --------------------------**WCSTCLDLACGASRE-CYDPCFKAFGRAHGKCMNNKCRCYT**------------- 40 29

sp|P0C185|KTx12.3_**TITCO** --------------------------**WCSTCLDLECGASRE-CYDPCFKAFGRAHGKCMNNKCRCYT**------------- 40 29

sp|P0C166|KTx6.12_**ANUPH**  -------------------------------**-QKECTGPQH-CTNFCRKNKCTHG-KCMNRKCKCFN-CK-**--------- 35 29

sp|P84864|KTx6.14_**HADGE**  -------------------------------**TGTSCISPKQ-CTEPCRAKGCKHG-KCMNRKCHCML-CL-**--------- 36 29

sp|P0C194|KTx6.11_**OPIMA**  ------MKVAYLLVLFTIMMLANDAS--LVHTNIPCRGTSD-CYEPCEKKYNCARAKCMNRHCNCYNNCPWR-------- 63 25

sp|C5J896|KTx6.16_**OPICY**  --MNLKLALVLLLTVINVGMLPGATSNGNIKTDIKCYRNSH-CNFHCEKSYYCQGSKCVRKRCNCYN-CPL--------- 67 23
