# Supplementary material for: The Mediterranean scorpion Mesobuthus gibbosus (Scorpiones, Buthidae): transcriptome analysis and organization of the genome encoding chlorotoxin-like peptides
Source: BMC Genomics. 2014 Apr 21;15:295. doi: 10.1186/1471-2164-15-295 (PMC4234519; doi:10.1186/1471-2164-15-295)
Supplement: Additional file 3: Figure S2 — Nucleotide sequences and deduced amino acid sequences of the Meg-chlorotoxins-like from M. gibbosus. Nucleotide sequences and the corresponding amino acid sequence deduced from gDNA. In the Genomic DNA sequences (gMeg) the exons are written in capital letters; the introns sequences are in lower and; highlighted letters show identical nucleotides in gray. Amino acid sequences (aMeg) show the predicted signal peptides underline; putative mature sequences are in bold; cysteines residues are highlighted in red. The alignment includes Bm12 gene from Mesobuthus martensii[34] and the amino acid sequence of the Chlorotoxin from Leiurus quinquestriatus quinquestriatus[32]. Most of the eukaryotic proteins-coding genes are interrupted by introns that are removed at the donor and acceptor splice sites such that the adjacent exons are spliced. Introns occur in three phases that are defined as the position of the intron within or between codons: intron of phases 0, 1 and 2 are located respectively, between two codons, after the first position in a codon, and after the second position [64]. MegClTxs introns have a consensus splice sites of gt at the 5’-end and ag at the 3’-end. Donor splice sites to exon-intron were conserved to the three MegClTxs genes (5’GTAATGATCG∧gtaagtgatt3’), showed phase 0 to MegClTx1 and MegClTx 3, while MegClTx2 was phase 2. Acceptor splice sites intron-exon was conserved to the three genes also (5’ccttttatag∧CAACTCATAC3’), all the genes showed phase 2. Bm12 gene has the same sequence to the donor splice sites than MegClTx2 and showed phase 2. However, it shows a different sequence to the acceptor splice sites phase 1 (atttatgtag∧CAACTCAAAC). [file 1471-2164-15-295-S3.doc]

## Figure S2

AYEL01091720.1|gBmKClTx1 ------------------------------------------------------------ 0

AYEL01055851.1|gBmKClTx5 ATGAAGTTCCTCTATGGAATCATTTT------------------------------taag 30

Q9UAD0.1|gBm12 ATGAAGTTCCTCTACGGAATCGTTTTCATTGCACTTTTTCTAACTGTAATGTTCGgtaag 60

KF743060|gMegClTx1 ATGAAGTTCCTCTATGGAATCGTTTTCATTACTCTTTTTTTAACAGTAATGATCGgtaag 60

KF743061|gMegClTx2 ATGAAGTTCCTCTATGGAATCGTTTTCATTGCTCTTTTTTTAACAGTAATGATCGgtaag 60

KF743062|gMegClTx3 ATGAAGTTCCTCTATGGAATCGTTTTCATTACTCTTTTTTTAACAGTAATGATCGgtaag 60

AYEL01091720.1|aBmKClTx1 - - - - - - - - - - - - - - - - - - - -  **0**

AYEL01055851.1|aBmKClTx5 M K F L Y G I I F - - - - - - - - - - -  **9**

Q9UAD0.1|aBm12 M K F L Y G I V F I A L F L T V M F - - **-18**

KF743060|aMegClTx1 M K F L Y G I V F I T L F L T V M I - - **-18**

KF743061|aMegClTx2 M K F L Y G I V F I A L F L T V M I - - **-18**

KF743062|aMegClTx3 M K F L Y G I V F I T L F L T V M I - - **-18**

P45639.1|aClTx - - - - - - - - - - - - - - - - - - - -  **0**

AYEL01091720.1|gBmKClTx1 ------------------------------------------------------------ 0

AYEL01055851.1|gBmKClTx5 tgattgtcaatatttatattaaagaatttaaaatcaataat*atgaaattaattttttatt* 90

Q9UAD0.1|gBm12 tggttgccaatatttatgttaaagaatttaaaatcaataatatgaaattaattt--tatt 118

KF743060|gMegClTx1 tgattgctaatatttatat---a--atttaaaatcaataatatgaaattaattttttatt 115

KF743061|gMegClTx2 tgattgttaatatttatat---agaatttaaaatcaataatatgaaattaattttttatt 117

KF743062|gMegClTx3 tgattgctaatatttatat---a--atttaaaatcaataatatgaaattaattttttatt 115

AYEL01091720.1|aBmKClTx1 - - - - - - - - - - - - - - - - - - - -  **0**

AYEL01055851.1|aBmKClTx5 - - - - - - - - - - - - - - *M K L I F Y*  **0**

Q9UAD0.1|aBm12 - - - - - - - - - - - - - - - - - - - - **-18**

KF743060|aMegClTx1 - - - - - - - - - - - - - - - - - - - - **-18**

KF743061|aMegClTx2 - - - - - - - - - - - - - - - - - - - - **-18**

KF743062|aMegClTx3 - - - - - - - - - - - - - - - - - - - - **-18**

P45639.1|aClTx - - - - - - - - - - - - - - - - - - - -  **0**

AYEL01091720.1|gBmKClTx1 --------------------------------------------------ATGTGTATGC 10

AYEL01055851.1|gBmKClTx5 *tcgtaataacatactattttctttctatag*CAACTCATTCTGAAGCT---ATGTGTATGC 145

Q9UAD0.1|gBm12 tagtaataacatattattttatttatgtagCAACTCAAACTGATGG------ATGTGGGC 172

KF743060|gMegClTx1 tcgtaataa-ac-ttattttccttttatagCAACTCATACTGAAGCTGCTATGTGTATGC 173

KF743061|gMegClTx2 ttgtaataa-ac-ttattttccttttatagCAACTCATACTGAAGCT---ATGTGTATGC 172

KF743062|gMegClTx3 ttgtaataa-ac-ttattttccttttatagCAACTCATACTGAAGCT---ATGTGTATGC 170

AYEL01091720.1|aBmKClTx1 - - - - - - - - - - - - - - - - - **M C M P +4**

AYEL01055851.1|aBmKClTx5 *F V I T Y Y F L S I* A T H S E A - **M C M P +4**

Q9UAD0.1|aBm12 - - - - - - - - - - A T Q T D G - **- C G P** **+3**

KF743060|aMegClTx1 - - - - - - - - - - A T H T E A A **M C M P +4**

KF743061|aMegClTx2 - - - - - - - - - - A T H T E A - **M C M P +4**

KF743062|aMegClTx3 - - - - - - - - - - A T H T E A - **M C M P +4**

P45639.1|aClTx - - - - - - - - - - - - - - - - - **M C M P +4**

AYEL01091720.1|gBmKClTx1 CTTGCTTTACAACGGATCCTAATATGGCAAGGAAATGTAGGGACTGTTGCGGAGGATATG 70

AYEL01055851.1|gBmKClTx5 CTTGCTTTACAATGGATCATAATATGGCAAAGAAATGTAGGGACTGTTGCAGAGGCAAGG 205

Q9UAD0.1|gBm12 CTTGCTTTACAACGGATGCTAATATGGCAAGGAAATGTAGGGAATGTTGCGGAGGTATTG 232

KF743060|gMegClTx1 CTTGCTTTACAACCAATCTTAATATGGAACAGGAGTGTAGGGACTGTTGCGGAGGCACGG 233

KF743061|gMegClTx2 CTTGCTTTACAACCCGTCCTAATATGGCACAGCAGTGTAGGGACTGTTGCAGAGGCAGGG 232

KF743062|gMegClTx3 CTTGCTTTACAACCCGTCCTAATATGGCACAGCAGTGTAGGGACTGTTGCAGAGGCAGGG 230

AYEL01091720.1|aBmKClTx1 **C F T T D P N M A R K C R D C C G G Y G +24**

AYEL01055851.1|aBmKClTx5 **C F T M D H N M A K K C R D C C R G K G +24**

Q9UAD0.1|aBm12 **C F T T D A N M A R K C R E C C G G I G +23**

KF743060|aMegClTx1 **C F T T N L N M E Q E C R D C C G G T G +24**

KF743061|aMegClTx2 **C F T T R P N M A Q Q C R D C C R G R G +24**

KF743062|aMegClTx3 **C F T T R P N M A Q Q C R D C C R G R G +24**

# P45639.1|aClTx C F T T D H Q M A R K C D D C C G G K G +24

AYEL01091720.1|gBmKClTx1 GAAAA------TGCTTTGACCCACAATGTCTGTGTGGCTATGAAtga 111

AYEL01055851.1|gBmKClTx5 GAAAA------TGCATTGGCCCACAATGTCTGTG--GCTATGGAtga 244

Q9UAD0.1|gBm12 GAAAA------TGCTTTGGCCCACAATGTCTGTGTAACCGTATAtga 273

KF743060|gMegClTx1 GAAGA------TGTTTTGGTCCACAATGTCTGTGTGGCTATGACtga 274

KF743061|gMegClTx2 GAAAA------TGTTTTGGTCCACAATGTCTGTGTGGCTATGACtga 273

KF743062|gMegClTx3 GAAAA------TGTTTTGGGCCACAATGTCTGTGTGGCTATGACtga 271

AYEL01091720.1|aBmKClTx1 **K - - C F D P Q C L C G Y E stop +36**

AYEL01055851.1|aBmKClTx5 **K - - C I G P Q C L C G Y G stop +36**

Q9UAD0.1|aBm12 **K - - C F G P Q C L C N R I** stop **+35**

KF743060|aMegClTx1 **R - - C F G P Q C L C G Y D** stop **+36**

KF743061|aMegClTx2 **K - - C F G P Q C L C G Y D** stop **+36**

KF743062|aMegClTx3 **K - - C F G P Q C L C G Y D** stop **+36**

P45639.1|aClTx **R G K C Y G P Q C L C R** - - **+36**
